# Understanding perioperative risk determinants in carotid endarterectomy: the impact of compromised circle of Willis morphology on inter-hemispheric blood flow indices based on intraoperative internal carotid artery stump pulse pressure and backflow patterns

**DOI:** 10.1007/s11357-024-01390-y

**Published:** 2024-10-26

**Authors:** Zsófia Czinege, Ágnes Dóra Sándor, Dániel Gyürki, Andrea Varga, Tamás Csípő, Andrea Székely, Zoltán Ungvári, Péter Banga, Péter Sótonyi, Tamás Horváth

**Affiliations:** 1 Department of Vascular and Endovascular Surgery, Semmelweis University, Budapest, 1122 Hungary; 2 Department of Anesthesiology and Intensive Therapy, Semmelweis University, Budapest, 1082 Hungary; 3 Department of Hydrodynamic Systems, Budapest University of Technology and Economics, Budapest, 1111 Hungary; 4 Department of Diagnostic Radiology, Heart and Vascular Center, Semmelweis University, Budapest, 1122 Hungary; 5 Institute of Preventive Medicine and Public Health, Semmelweis University, Budapest, Hungary; 6 Vascular Cognitive Impairment, Neurodegeneration and Healthy Brain Aging Program, Department of Neurosurgery, University of Oklahoma Health Sciences Center, Oklahoma City, OK USA; 7 International Training Program in Geroscience, Doctoral College/Institute of Preventive Medicine and Public Health, Semmelweis University, Budapest, Hungary; 8 Research Center for Sport Physiology, Hungarian University of Sports Science, Budapest, 1123 Hungary

**Keywords:** Collateral, Shunt, Carotid artery stenosis, Compensation, Complication, Surgery, Aging, Circle of Willis

## Abstract

Carotid artery stenosis (CAS) often requires surgical intervention through carotid endarterectomy (CEA) to prevent stroke. Accurate cerebrovascular risk assessments are crucial in CEA, as poor collateral circulation can lead to insufficient interhemispheric blood flow compensation, resulting in ischemic complications. Therefore, understanding perioperative risk determinants is vital. This study aims to determine the impact of compromised circle of Willis (CoW) morphology on inter-hemispheric blood flow, focusing on indices based on intraoperative internal carotid artery stump pulse pressure and backflow patterns. In 80 CAS patients who underwent CEA, preoperative CT angiography for CoW was conducted. Patients were categorized into five subgroups based on their CoW anatomy and three additional groups based on intraoperative internal carotid artery (ICA) stump backflow patterns evaluated by the surgeon. Continuous blood pressure signals, including systolic, diastolic, mean, and pulse pressure values, were recorded during the procedure. The relationship between CoW anatomical variants and the systolic and diastolic segments of the averaged pressure waveforms, particularly diastolic pressure decay, was analyzed. The correlation between CoW anatomy and stump backflow intensity was also examined. Significant variability in ICA stump backflow and pressure values was evident across CoW variants. Patients with compromised CoW morphology exhibited weaker backflow patterns and lower ICA stump pulse pressure values, consistent with impaired interhemispheric blood flow. Notably, ICA stump diastolic pressure decay was consistent across most CoW variant groups, indicating developed collateral circulation in cases with CoW anomalies. Thus, impaired CoW integrity is associated with compromised interhemispheric blood flow indices based on intraoperative ICA stump pulse pressure and backflow patterns during CEA. Integrating intraoperative pulse waveform analysis with preoperative CT angiography provides a more detailed assessment of cerebrovascular risk, guiding the selective use of shunts. This combined approach may improve surgical outcomes and patient safety by identifying patients at increased risk of perioperative neurological events due to CoW anomalies.

## Introduction

Internal carotid artery stenosis (CAS) is a steno-occlusive condition resulting from atherosclerosis, which is linked to unhealthy vascular aging [1]. In older adults, the prevalence of CAS ranges from approximately 5 to 7.5% [2–4]. The narrowing of the internal carotid artery (ICA) due to atherosclerotic plaque can lead to thromboembolism, causing ischemic events (transient ischemic attack [TIA] or ischemic stroke) in the brain [5]. Atherosclerotic CAS is a significant risk factor for ischemic stroke, a major cause of death and long-term disability in developed countries [6–8]. To prevent stroke, patients with substantial CAS are typically treated with either eversion endarterectomy or endarterectomy with patch plasty [1, 9].

Carotid endarterectomy (CEA) is a surgical procedure that involves temporarily interrupting cerebral blood flow to remove atherosclerotic plaque from the carotid artery. Perioperative complications during CEA can include ischemic stroke, transient ischemic attacks, and other neurological deficits. These complications are influenced by several risk factors, including the morphological, structural, and functional integrity of the cerebral circulation [5, 10–27]. Adequate hemodynamic compensation during CEA is crucial to minimize medical and surgical complications. The effectiveness of interhemispheric blood flow compensation plays a critical role in mitigating these risks. Effective compensation depends on several factors.

The presence and robustness of collateral pathways are crucial. These vessels can provide alternative routes for blood flow when the primary pathways are obstructed. The structural configuration of the CoW significantly impacts interhemispheric blood flow compensation [15, 19, 20, 28–38]. A complete and well-formed CoW can provide redundant pathways for blood flow, reducing the risk of ischemia during carotid occlusion. Conversely, anatomical variations or incomplete CoW formations can limit these compensatory mechanisms, increasing vulnerability to ischemic events. Additionally, collateral circulation plays a vital role, including extracranial-intracranial pathways such as the ophthalmic artery and middle meningeal artery, intracranial pathways like the pial branches of cerebral arteries and leptomeningeal collaterals, and skull base perforators such as the lenticulostriate and thalamoperforating branches [39]. These collaterals enhance the brain’s ability to compensate for reduced perfusion during CEA, contributing to the overall stability of cerebral blood flow [12–27].

The cerebral autoregulatory capacity, which allows resistance vessels to dilate in response to a drop in perfusion pressure, is essential. This dilation helps maintain adequate cerebral blood flow during interruptions like those that occur in CEA. Effective autoregulation is complemented by endothelium-mediated vasodilation, which significantly reduces cerebral vascular resistance. Understanding these factors is vital for assessing perioperative risk and planning interventions such as selective shunting, which can help maintain cerebral perfusion and reduce the likelihood of neurological complications during CEA [40, 41].

Recent guidelines recommend that the decision to use shunting during CEA should be made by the surgeon [42]. This decision is influenced by the patient’s risk factors and medical history, particularly the presence of contralateral carotid occlusion or significant contralateral carotid stenosis [43, 44]. Additionally, there is a need for an objective, comprehensive, and reliable monitoring approach to identify patients at risk of developing neurological events during the perioperative period.

Information on the effectiveness of interhemispheric blood flow compensation is crucial. For instance, precise preoperative imaging of CoW anatomies aids in shunt decisions, as certain configurations are linked to increased perioperative neurological risks [45–47]. However, identifying other collateral networks preoperatively is complex due to the lack of standardized imaging techniques [48–50]. Advanced modalities like digital subtraction angiography (DSA), transcranial Doppler sonography (TCD), computer tomography (CT), and magnetic resonance (MR) perfusion imaging are used for collateral assessment, with DSA being the benchmark technique [51–54]. Intraoperative monitoring during CEA supports shunting decisions and includes awake neurologic assessment, near-infrared spectroscopy (NIRS), electroencephalography (EEG), continuous TCD, and somatosensory evoked potentials (SSEP) [55–65]. However, relying solely on one monitoring method is not recommended. The presence of neurological symptoms before CEA can also provide additional information for shunting decisions.

Stump pressure measurement is a pivotal technique used during CEA to evaluate cerebral perfusion pressure when the carotid artery is clamped [61–65]. This method involves measuring the blood pressure distal to the occlusion site, providing an indirect assessment of the collateral blood flow to the brain. A higher stump pressure indicates adequate collateral circulation and a lower risk of cerebral ischemia during the procedure. Conversely, a low stump pressure suggests insufficient collateral blood flow, which may necessitate the use of a shunt to maintain cerebral perfusion.

Building on this, our study aims to characterize the impact of compromised CoW morphology and evaluate collateral circulation based on patients’ unique central arterial pulse waveforms. We examined the association between CoW morphology, the surgeon’s semi-quantitative evaluation of ICA backflow, and performed ICA stump pressure waveform analysis in asymptomatic carotid disease patients. Pressure waveform analysis quantifies the time-course and morphology of recorded systolic and diastolic blood pressure values. An important aspect of this analysis is the diastolic pressure decay, characterized by the decay time constant (τ). According to simple Windkessel models [66], τ equals peripheral resistance (*R*) multiplied by total compliance (*C*) (τ = *R* × *C*). The decay time constant is a critical parameter in understanding the hemodynamics of the cerebral circulation. It reflects the interplay between vascular resistance and total arterial compliance, providing insight into the capacity of blood vessels to accommodate and transmit pulsatile blood flow. Biologically, a longer τ indicates greater compliance and lower vascular resistance, suggesting a more resilient vascular system capable of buffering changes in blood pressure and maintaining stable blood flow. Conversely, a shorter τ implies higher resistance and lower compliance, which can lead to increased hemodynamic stress on the cerebral vasculature and a greater propensity for ischemic events during conditions of reduced perfusion, such as during carotid endarterectomy. Therefore, measuring τ can help assess the efficiency of cerebral blood flow regulation and the capacity of the cerebral arteries to compensate for interruptions in blood flow. In addition, backflow measurement, which assesses the blood flow returning through the ICA when clamped, is also investigated in relation to CoW anatomy. Both stump pressure waveform indices and backflow are relevant for understanding the effectiveness of interhemispheric blood flow compensation. Our study examines how variations in CoW anatomy impact both stump pressure waveform characteristics and backflow patterns, providing a comprehensive assessment of cerebral perfusion dynamics during CEA. This dual approach enhances our ability to predict and manage perioperative neurological risks effectively.

## Methods

### Study participants

This study received ethical approval from the appropriate institutional review board (IRB) by the Semmelweis University Regional and Institutional Committee of Science and Research Ethics (IV/667–1/2022/EKU) and was carried out in accordance with the Declaration of Helsinki (SE-KREB 84/2019). We prospectively collected data from 80 consecutive patients who underwent carotid endarterectomy (CEA) between May 1, 2019, and October 31, 2021. All participants provided written consent after receiving a thorough explanation of the study. Participation was offered to patients diagnosed with significant (> 70%) carotid artery stenosis confirmed by ambulatory CT angiography (CTA) scans, who provided a signed consent form and met the study eligibility criteria. Exclusion criteria included age below 50 years, non-compliance, presence of manifested neurological symptoms (amaurosis fugax, TIA, stroke) within 15 days, presence of a pacemaker or ICD (implantable cardioverter defibrillator), atrial fibrillation, and/or chronic kidney disease (stage V).

Data regarding demographic, clinical, imaging, and procedural details were collected. Hypertension was defined according to the American Heart Association guidelines [67], while diabetes and dyslipidemia were defined according to the European Society of Cardiology’s standards [68]. Following the current guidelines of the European Society of Vascular Surgery, optimal medicinal therapy consisting of either aspirin (100 mg) or clopidogrel (75 mg) in conjunction with a statin was administered based on the CTA results. Symptomatic carotid artery stenosis (relating to a significant neurological episode within the previous 6 months) and asymptomatic carotid artery stenosis were determined in accordance with the European Society of Vascular Surgery’s guidelines [42]. Asymptomatic patients with greater than 70% stenosis underwent carotid reconstruction in accordance with the North American Symptomatic Carotid Endarterectomy Trial (NASCET) criteria [69].

### Computed tomography angiography and preoperative assessment of supra-aortic arteries and the CoW

CTA was utilized for the preoperative assessment of supra-aortic arteries and the CoW in patients scheduled for CEA. CTA provided detailed imaging that allowed for the evaluation of the anatomical structure and patency of the supra-aortic arteries, as well as the configuration and integrity of the CoW. This comprehensive assessment was crucial in identifying variations in CoW anatomy, which could influence interhemispheric blood flow compensation and perioperative risk. The detailed visualization afforded by CTA enabled precise planning for the surgical approach and informed decisions regarding the necessity of intraoperative shunting based on the presence of collateral pathways and potential vulnerabilities in cerebral perfusion.

For assessment purposes, an experienced radiologist, blinded to patient characteristics and clinical outcomes, analyzed the carotid and vertebral arteries, as well as the morphology of the CoW. This evaluation was conducted using a specialized workstation (IntelliSpace Portal, Philips Healthcare, Best, The Netherlands). The study employed the NASCET criteria [69], utilizing vendor-supplied software (Advanced Vascular Analysis, Philips Healthcare) to calculate the percentage of internal carotid artery (ICA) stenosis. Detailed analysis of the CoW’s anatomical structure was performed, focusing on the anterior communicating artery (A-com), both first segments of the anterior cerebral arteries (A1), the posterior communicating arteries (P-com), and the first segments of the posterior cerebral arteries (P1). These components were classified as normal if their diameter was 0.8 mm or greater, hypoplastic if visible but less than 0.8 mm in diameter, and absent if not visualized. The anterior semicircle of the CoW consists of the contralateral A1, the A-com, and the ipsilateral A1, while the posterior semicircle is formed by the P1 and P-com on each side [46]. Patients were subsequently grouped into five subgroups based on the anatomical configuration of the CoW:

- Complete CoW: All segments are normal.
- Compromised anterior CoW: One of the anterior CoW segments is absent/non-visualized or hypoplastic.
- Compromised posterior CoW: One of the posterior CoW segments is absent/non-visualized or hypoplastic.
- Both anterior and posterior CoW Compromised: Hypoplastic-hypoplastic segments, absent/non-visualized-hypoplastic segments, or hypoplastic-absent/non-visualized segments in the anterior and posterior semicircles.
- Isolated middle cerebral artery (MCA): Absent/non-visualized segments in both the anterior and posterior semicircles, with the MCA on the operated side not receiving primary collateral flow from either the contralateral ICA or the vertebrobasilar system.

We also evaluated the degree of contralateral carotid stenosis, which allowed us to investigate its impact on ICA pressure waveforms during CEA. This comprehensive analysis provided insights into the relationship between CoW anatomy, collateral circulation, and cerebral perfusion dynamics during carotid artery surgery.

### MRI imaging methodology

Non-contrast brain MRI scans were conducted on patients undergoing CEA both pre-operatively (12–24 h prior) and post-operatively (12–48 h following the procedure). These scans were performed using a Siemens Magneton Aera 1.5 T scanner, equipped with standard head array coils. The MRI protocol included diffusion tensor imaging (DTI), FLAIR, T2*-gradient echo, and susceptibility weighted imaging (SWI). The primary objective of the MRI evaluation was to identify and characterize acute ischemic lesions, providing critical information on cerebral perfusion status and potential perioperative complications.

### Operation technique, carotid artery eversion endarterectomy and backflow assessment

All surgical procedures were performed under general anesthesia, with the desired mean arterial pressure maintained at 80 mmHg or greater following systemic heparin administration. The common carotid artery (CCA), external carotid artery (ECA), and ICA were prepared, and a clamp was placed on the artery to access the built-up plaque within the ICA lumen. Clamp time, measured from the application to the removal of the carotid clamp, averaged 28.5 ± 9.8 min in our patient group. The plaque was then removed from the ICA using an eversion technique.

After plaque removal, the operator recorded ICA stump pressure waveforms for 60 s using a plastic cannula inserted into the lumen, ensuring the distal endpoint did not exceed the base of the skull. Subsequently, each surgeon subjectively evaluated ICA stump blood backflow while systemic mean blood pressure was kept at 80 mmHg. Based on stump backflow, patients were classified into three subgroups:

- Backflow 1: Weak and discontinuous
- Backflow 2: Continuous but not pulsatile
- Backflow 3: Strong and pulsatile

Following hemodynamic data collection, the carotid bulb and the proximal portion of the external carotid artery were reconstructed. If the carotid lesion was too high for eversion or the patient had a compromised anatomical variation of the CoW, the operation was performed under shunt protection. Shunting was utilized infrequently (*n* = 22, 27.5%) and was at the discretion of the surgeon. In our study, the average duration of shunting was 38.3 ± 14.6 min.

### Stump pressure waveform analysis and data acquisition

Invasive systemic blood pressure monitoring was conducted via the contralateral radial artery. A saline-filled arterial line was connected to a Braun Combitrans disposable blood pressure transducer through a three-way stopcock. This setup transmitted pressure-analog electronic signals to a GE Datex Ohmeda Aisys CS2 patient monitor. For stump pressure measurement, the stopcock was turned to connect the transducer to a plastic cannula introduced into the ICA stump. The Datex-Ohmeda S/5 Collect software (GE Healthcare USA) digitized and recorded both radial and carotid pressures at a sampling rate of 100 Hz, ensuring high-resolution data acquisition for detailed waveform analysis.

### Stump pressure waveform data processing

Recorded time-series data were exported to text files for offline processing using custom-written Python scripts (Python 3.12.0). Additionally, we incorporated and adapted a specialized Python package designed for cerebrovascular pulse waveform analysis, enhancing its capability to process and interpret the unique pulse wave characteristics pertinent to our research objectives. This bespoke integration of the pulse wave analyzing package with our Python scripts facilitated a tailored approach to our cerebrovascular data analysis needs. Signals were low-pass filtered at 10 Hz, and a second-order Savitzky-Golay polynomial smoothing filter [70] (window size = 50 ms) was applied to the raw pressure data.

After mapping out all the cardiac cycles, a two-step signal averaging was conducted to ensure data accuracy. Each pressure cycle was aligned by its fiducial points for signal averaging. In the absence of simultaneously recorded ECG signals, fiducials were defined as the point at the maximum of the second derivative on the anacrotic pressure limb. The first line averaging resulted in a “raw average,” then each individual pressure cycle was correlated against the “raw average,” and only those waveforms with a correlation coefficient above 0.95 were considered for the final analysis. Cycles that passed this criterion were re-averaged to obtain a representative pressure waveform. Pulse wave morphological analysis was carried out on these pressure data.

**Fig. 1:**
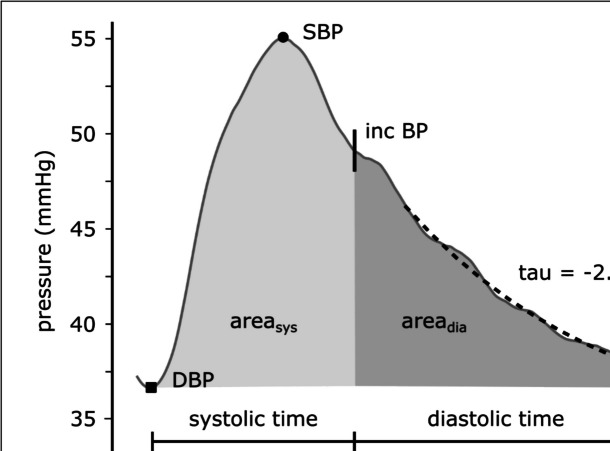
Blood pressure waveform analysis. SBP, DBP, and incBP are standing for systolic, diastolic, and end-systolic blood pressure values. Systolic and diastolic areas are depicted in light and darker grey. Diastolic pressure decay time constant calculation based on the area method (grey filled diastolic area)

During waveform analysis, amplitude values, time durations, and area values were calculated. Measurements included systolic, diastolic, pulse, and mean pressures, as well as the end-systolic (dicrotic notch) pressure. Systolic and diastolic time durations were calculated, and the total, systolic, and diastolic areas were derived. The diastolic pressure decay time constant (tau) was calculated from the area under the pressure curve, due to its superior robustness and noise tolerance compared to other methods [71–73]. Pulse wave analysis parameters are illustrated in Fig. 1.

### Statistical analysis

Statistical analysis was performed using R (version 4.2.2). Chi-square or Fisher’s exact tests were used to compare clinical categorical variables. The influence of systemic blood pressure on ICA pressure was analyzed by creating ICA/systemic blood pressure ratio variables. Normal distribution of data was determined based on visual inspection of Q-Q plots of each continuous variable. One-way ANOVA tests, with CoW classification as the group variable, were used to assess each pulse waveform parameter. This was followed by pairwise Tukey tests for mean comparisons between CoW variants. The relationship between the grade of contralateral carotid stenosis and pulse waveform parameters was tested using Pearson correlation, while Spearman correlation assessed the link between backflow intensity and CoW classification. A p-value of < 0.05 was considered statistically significant.

## Results

### Clinical data, pre-, post-, and perioperative neurological status, risk factors

Out of 80 patients, 2 were excluded due to poor pressure data quality, leaving 78 patients (31 women and 47 men) with an average age of 69.6 ± 7.2 years included in this study. In terms of categorical clinical data, patient history, comorbidities, and medications, there were no significant differences among the patient groups with different CoW variants (Table 1).

**Table 1:** Clinical, hemodynamical anthropometrical characteristics of the study sample

Variables	CoW 1	CoW 2	CoW 3	CoW 4	CoW 5	All patients	*p* value
Anthropometric variables							
*n*	12	12	31	18	5	78	
Age (years)	66.8 ± 6.4	70.0 ± 8.6	71.1 ± 6.9	69.9 ± 6.4	65.2 ± 8.9	69.6 ± 7.2	NS
Sex (male)	8	4	20	10	5	47	NS
Weight (kg)	77.8 ± 15.0	81.2 ± 14.1	81.7 ± 17.5	78.3 ± 10.4	85.6 ± 21.2	80.5 ± 15.2	NS
Height (cm)	1.69 ± 0.1	1.68 ± 0.1	1.7 ± 0.08	1.66 ± 0.06	1.74 ± 0.08	1.69 ± 0.08	NS
BMI (kg/m^2^)	26.9 ± 4.1	29.2 ± 5.2	28.4 ± 6.1	28.7 ± 3.4	28.0 ± 5.2	28.4 ± 5.02	NS
Brachial cuff blood pressures							
SBP left (mmHg)	144.0 ± 25.4	150.0 ± 15.5	140.0 ± 19.1	148.0 ± 18.9	127.0 ± 27.5	143.1 ± 20.5	NS
DBP left (mmHg)	80.2 ± 10.2	81.9 ± 15.4	80.7 ± 13.0	80.4 ± 10.1	77.6 ± 19.1	81.4 ± 12.7	NS
SBP right (mmHg)	140.0 ± 26.2	145.0 ± 23.1	144.0 ± 21.3	147.0 ± 21.0	129.0 ± 22.9	143.0 ± 22.2	NS
DBP right (mmHg)	80.2 ± 10.2	81.9 ± 15.4	80.7 ± 13.0	80.4 ± 10.1	77.6 ± 19.1	81.4 ± 12.7	NS
Clinical data and risk factors							
Preoperative neurological event	0	0	1	2	0	3	NS
Stroke	0	0	1	0	0	1	NS
TIA	0	0	0	2	0	2	NS
Amaurosis fugax	0	0	0	0	0	0	-
Postoperative neurological event	0	0	0	0	0	0	-
Postoperative acute ischemic lesions	1	2	5	0	1	9	NS
Alcohol	1	0	1	1	0	3	NS
Smoking	3	3	7	5	1	19	NS
HT	11	12	31	17	5	76	NS
DM	5	3	11	8	4	31	NS
Cardiac ischemia	6	1	6	4	1	18	NS
Medications							
ASA	9	8	14	10	3	44	NS
Clopidogrel	6	5	14	6	2	33	NS
Statin	6	11	26	14	4	61	NS

*CoW* circle of Willis, *CoW 1* CoW is complete, *CoW 2* anterior CoW is compromised, *CoW 3* posterior CoW is compromised, *CoW 4* anterior and posterior CoW is compromised, *CoW 5* medial cerebral artery is isolated, *BMI* body mass index, *SBP*, *DBP* brachial artery systolic and diastolic blood pressure, *TIA* transient ischemic attack, *HT* hypertension, *DM* diabetes mellitus, *ASA* acetylsalicylic acid

Carotid endarterectomies were performed under shunt protection in 24 cases. Shunting preference was significantly influenced by prior knowledge of CoW configuration, resulting in varying shunt use across CoW groups (*p* = 0.001). Shunt application was most frequent in cases with compromised posterior semicircles (CoW3; 9 out of 31 patients, 29%), both anterior and posterior semicircles compromised (CoW4; 8 out of 18 patients, 44%), and all isolated MCA cases (CoW5; 5 out of 5 patients, 100%).

During the postoperative period, no major neurological complications such as stroke, transient ischemic attack (TIA), or amaurosis fugax were detected. However, two cases experienced minor neurological incidents, specifically temporary difficulty in awakening. Additionally, silent acute ischemic lesions were observed in nine cases based on postoperative MRI. Despite these observations, no significant difference in the occurrence of ischemic lesions was found among the different CoW anatomy groups.

**Fig. 2:**
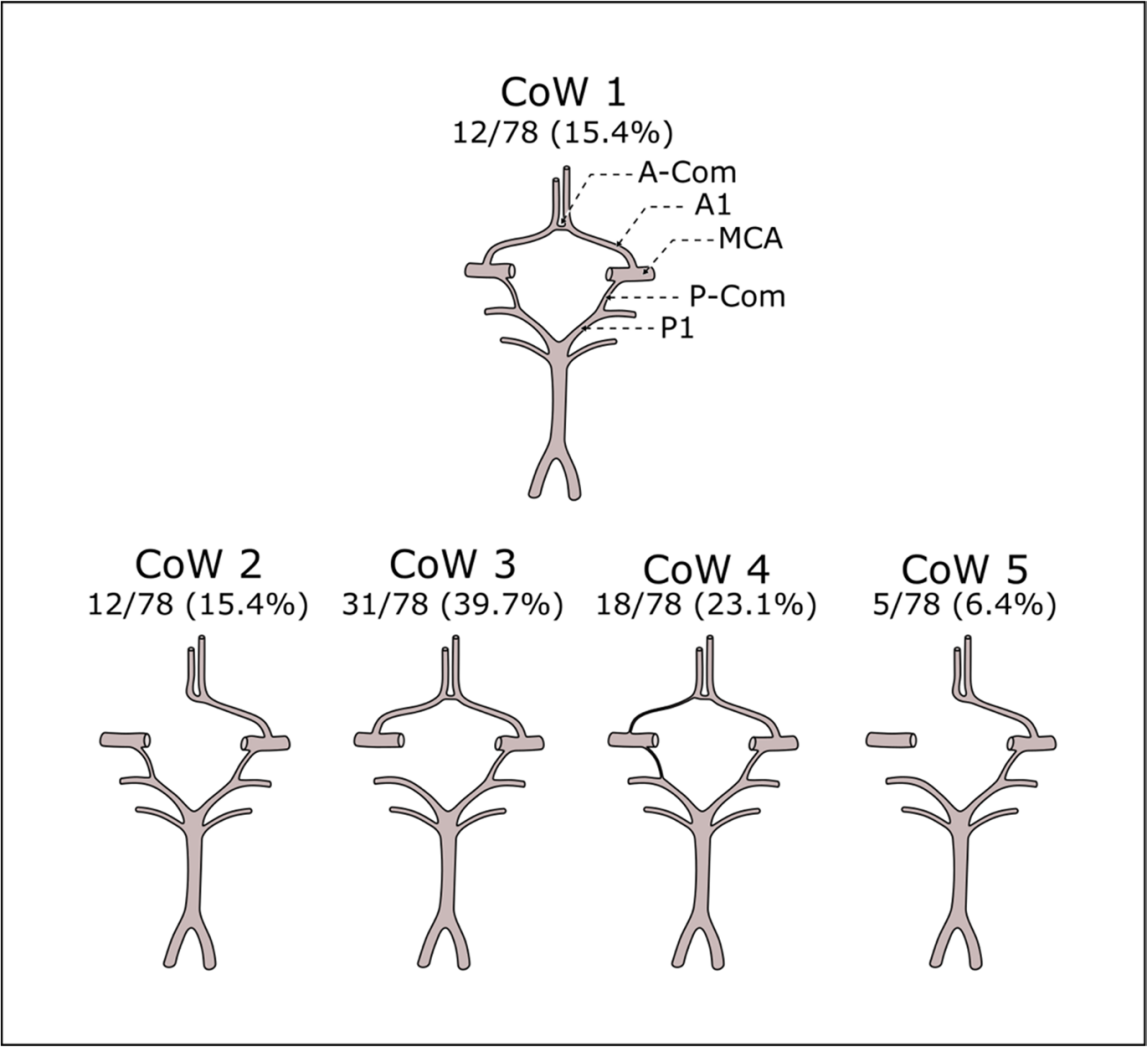
Circle of Willis variants. CoW 1: complete arterial circle; CoW 2: compromised anterior circle; CoW 3: compromised posterior circle; CoW 4: both anterior and posterior semicircles compromised; CoW 5: isolated middle cerebral artery. A-Com: anterior communicating artery; A1: first segment of the anterior cerebral artery; MCA: middle cerebral artery; P-Com: posterior communicating artery; P1: first segment of the posterior cerebral artery. Relative frequencies and percentages of different CoW variants displayed

Clinical and anthropometrical data are summarized in Table 1, and the frequencies of CoW variants are displayed in Fig. 2.

### Carotid stump backflow evaluation by the surgeon

**Fig. 3:**
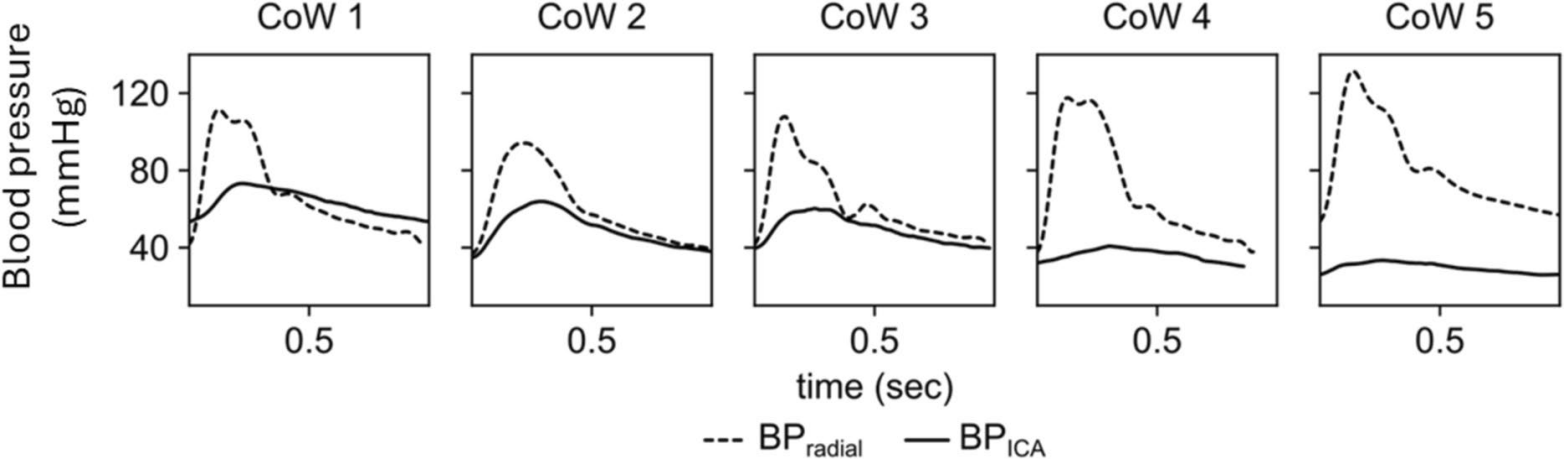
Representative blood pressure traces from patients with a complete circle of Willis (CoW 1) and different degrees of CoW impairment (CoW 2–5). Cow 1: complete CoW patient’s similar internal carotid (ICA) and radial artery (RAD) diastolic pressures, with marked pulse pressure difference due to ICA stenosis. CoW 2: compromised anterior circle; CoW 3: compromised posterior circle; CoW 4: both anterior and posterior semicircles compromised; CoW 5: isolated middle cerebral artery.BP_ICA_ and BP_radial_ stand for internal carotid and radial artery blood pressures

**Fig. 4:**
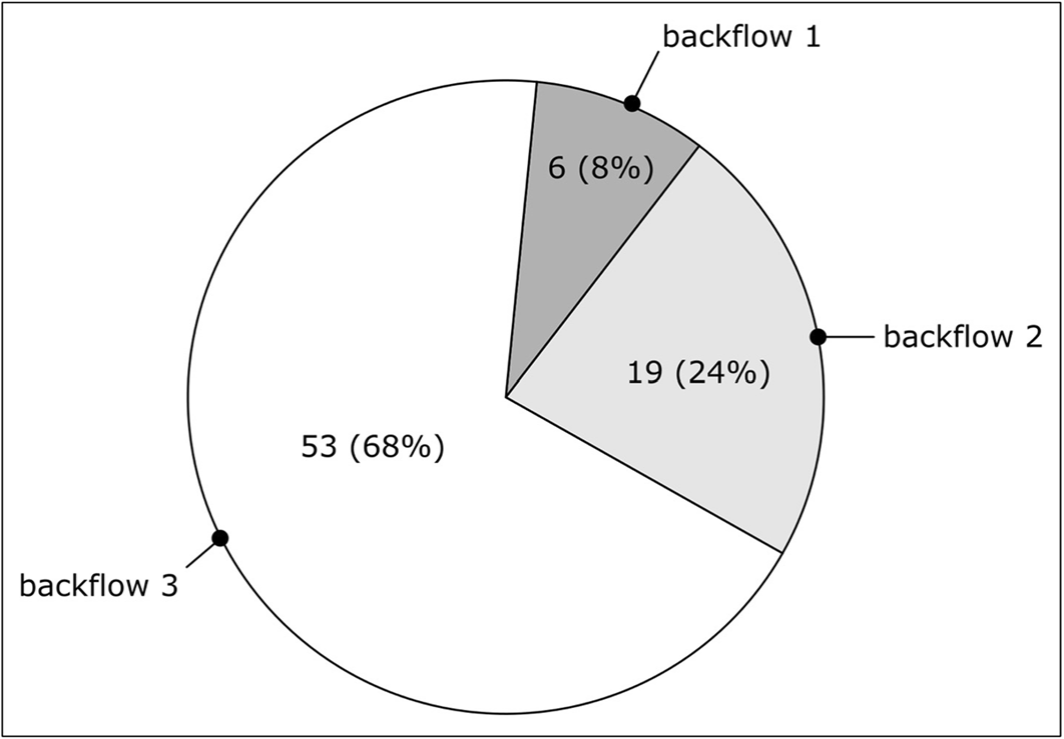
Carotid stump backflow groups. Frequencies in different ICA stump backflow intensity groups. Patients were assigned into 3 groups: backflow1—weak discontinuous; backflow2—continuous but not pulsatile; backflow3—strong pulsatile

Surgeons classified the ICA stump backflow patterns as strong pulsatile (53 cases, 68%), continuous but not pulsatile (19 cases, 24%), and weak discontinuous (6 cases, 8%). These on-site evaluations of backflow showed a moderate correlation with preoperative imaging information regarding the CoW anatomy (Spearman correlation coefficient, 0.37). The association between backflow classification and CoW anatomy is displayed in Table 2, where the numbers and colors reflect the number of patients in each group. Low backflow classifications (backflow 1) were more frequent in patients with compromised CoW circulations (CoW 3–5), whereas high backflow (backflow 3) was more commonly associated with less compromised or complete vascular morphologies (CoW 1–3) (Figs. 3 and 4).

**Table 2:**
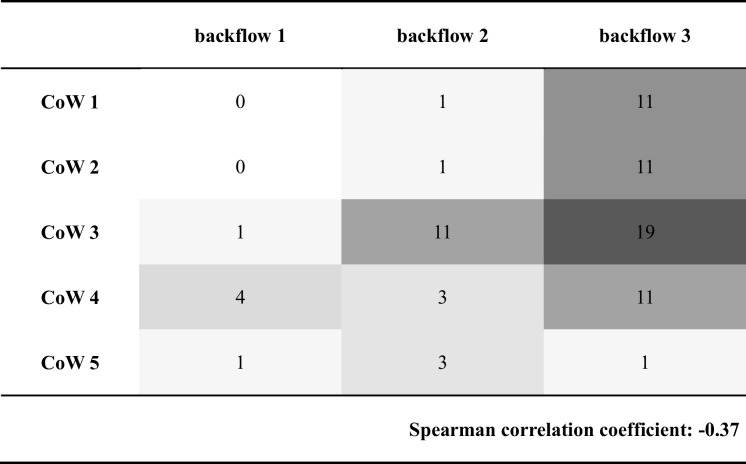
Internal carotid artery (ICA) backflow grouping and CoW grouping

Numbers and coloring indicate the frequency of the occurrences within each group. Column headers: backflow 1—weak, discontinuous; backflow 2—continuous but not pulsatile; backflow 3—strong pulsatile; row headers: *CoW* circle of Willis, *CoW 1* CoW is complete, *CoW 2* anterior CoW is compromised, *CoW 3* posterior CoW is compromised, *CoW 4* anterior and posterior CoW is compromised, *CoW 5* medial cerebral artery is isolated. It is noteworthy that CoW 2 and 3 patients had either a compromised anterior or posterior CoW; however, compromised compromised posterior CoW morphology was associated to a higher frequency of impaired ICA backflow

### Pulse pressure waveform variables

**Fig. 5:**
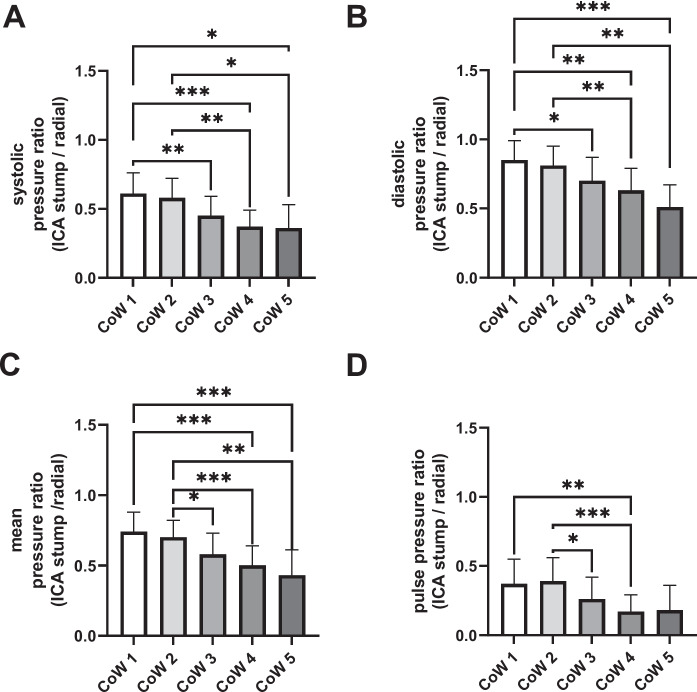
Internal carotid and radial blood pressure ratios in different circle of Willis (CoW) variants. Significant post hoc Tukey’s comparisons are marked with horizontal bars (*p < 0.05, **p < 0.01, ***p < 0.001). CoW 1: complete arterial circle; CoW 2: anterior compromised circle; CoW 3: posterior compromised circle; CoW 4: both anterior and posterior semicircles compromised; CoW 5: isolated middle cerebral artery. Data and error bars are displayed as mean and SD

**Fig. 6:**
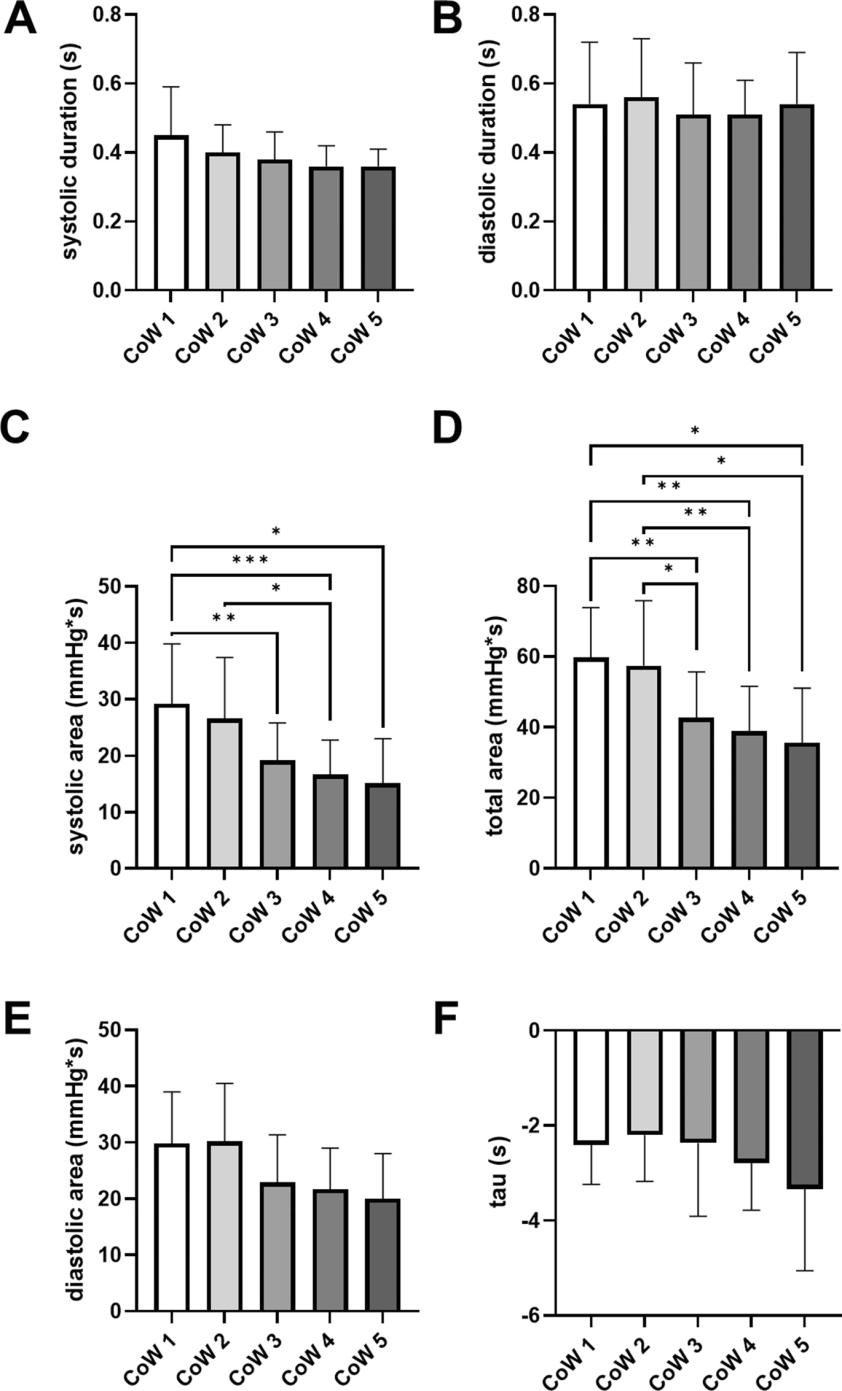
Pulse waveform analysis parameters in different circle of Willis (CoW) variants. Significant post hoc Tukey’s comparisons are marked with horizontal bars (*p < 0.05, **p < 0.01, ***p < 0.001). CoW 1: complete arterial circle; CoW 2: anterior compromised circle; CoW 3: posterior compromised circle; CoW 4: both anterior and posterior semicircles compromised; CoW 5: isolated middle cerebral artery. Data and error bars are displayed as mean and SD

There was no significant difference in average heart rates and systemic blood pressure values, as characterized by radial artery pressure, between the CoW groups, indicating comparable systemic hemodynamic status among our patients during anesthesia. However, there was a significant effect of CoW group on both raw blood pressure values in the ICA (Table 3). One-way ANOVA found a significant effect of CoW group on the stump pressure/radial pressure ratio values (systolic: *F* = 4.55 *p* = 0.002; diastolic:* F* = 3.756, *p* = 0.008; pulse pressure: *F* = 3.84, *p* = 0.007; mean: *F* = 3.11, p = 0.02). Raw pressures and pressure ratios displayed a decreasing trend from complete CoW cases toward the isolated MCA variants, as shown in Table 3 and Figs. 5 and 6. Pairwise post hoc Tukey’s test confirmed significant differences between several CoW groups: CoW 1 and 3, CoW 1 and 4, CoW 1 and 5, CoW 2 and 4, and CoW 2 and 5 (Fig. 5).

**Table 3:** Hemodynamic variables in CoW groups

	CoW 1	CoW 2	CoW 3	CoW 4	CoW 5	*p*-value
Internal carotid artery stump pressure						
SBP (mmHg)	74.8 ± 23.4	78.4 ± 31.8	59.5 ± 18.8	51.1 ± 16.4	46.7 ± 25.2	0.002
DBP (mmHg)	51.5 ± 10.6	50.7 ± 19.0	40.7 ± 10.8	38.9 ± 10.2	35.0 ± 15.9	0.008
PP (mmHg)	23.4 ± 15.1	27.7 ± 15.3	18.9 ± 11.4	12.2 ± 8.2^#^	11.7 ± 9.9	0.007
MBP (mmHg)	60.7 ± 15.7	59.8 ± 24.6	49.8 ± 13.6	44.9 ± 13.1	40.5 ± 20.1	0.02
incBP (mmHg)	62.1 ± 15.5	63.1 ± 24.4	49.2 ± 13.9	44.9 ± 12.7	40.5 ± 20.1	0.004
Radial artery pressure						
SBP (mmHg)	124.0 ± 29.3	135.0 ± 36.1	133.0 ± 23.9	140.0 ± 22.3	139.0 ± 27.4	NS
DBP (mmHg)	61.8 ± 16.5	62.3 ± 19.6	59.4 ± 12.0	63.6 ± 12.9	68.2 ± 14.1	NS
PP (mmHg)	62.3 ± 16.0	72.5 ± 22.0	73.2 ± 19.7	76.4 ± 14.4	70.4 ± 19.6	NS
MBP (mmHg)	85.3 ± 22.0	89.4 ± 26.8	86.0 ± 15.0	91.9 ± 16.1	93.9 ± 19.9	NS
incBP (mmHg)	84.9 ± 20.0	85.8 ± 22.7	82.1 ± 16.1	88.3 ± 17.0	92.9 ± 18.0	NS
HR (1/min)	63.4 ± 15.0	66.0 ± 17.5	70.2 ± 12.4	70.3 ± 12.0	68.4 ± 12.8	NS

Under comparable systemic cardiovascular status, ICA stump blood pressure differs in the CoW groups. SBP, DBP, PP, MBP, and incBP are standing for systolic, diastolic, pulse pressure, mean and incisura blood pressure; *tau* diastolic decay time constant, *HR* heart rate. Data presented as mean ± SD, *p* values are reported for one-way ANOVA testing. Results of post hoc Tukey’s tests are shown: *, significant difference from CoW 1; #, significant difference from CoW 2. No pairwise comparisons found significant differences between CoW 1 and 2, CoW 1 and 3, CoW 1 and 5, CoW 2 and 3, CoW 2 and 5, CoW 3 and 4, CoW 3 and 5, and CoW 4 and 5 groups

Although CoW group had no significant effect on systolic and diastolic durations (systolic: *F* = 2.26, *p* = 0.07; diastolic: *F* = 0.33, *p* = 0.85), one-way ANOVA detected a significant effect of CoW group on systolic (*F* = 6.91, *p* < 0.001), diastolic (*F* = 3.53 *p* = 0.01), and total (*F* = 6.98, *p* < 0.001) areas under the pressure curve. Post hoc pairwise testing confirmed that hypoplastic/isolated CoW variants displayed significantly lower systolic and total areas under the pressure curves (Fig. 6). The CoW group had no effect on the rate of diastolic pressure dissipation, quantified by tau values (*F* = 1.04, *p* = 0.39).

### Contralateral carotid artery pathology

The relationship between the severity of contralateral carotid stenosis and pulse waveform variables was weak, as indicated by Pearson correlation coefficients of less than 0.24 for all variables.

## Discussion

This study aimed to investigate the impact of CoW morphology on intraoperative cerebral hemodynamics during CEA by analyzing ICA stump pressure waveforms and backflow patterns. Our findings indicate that the configuration of the CoW significantly influences numerous ICA stump pressure waveform metrics, including systolic, diastolic, pulse, mean, and end-diastolic blood pressure values, which are critical for understanding the effectiveness of interhemispheric blood flow compensation.

We observed that patients with compromised CoW anatomy, particularly those with hypoplastic or absent segments, exhibited lower stump pressures and pressure ratios. The highest pressure values were measured in patients with a complete CoW and those with deficiencies confined to the anterior part of the CoW. A clear decreasing trend in stump pressures was noted in cases where posterior circulation deficiencies were evident on radiographic findings. Notably, patients with isolated MCA configurations exhibited the lowest stump pressure values within our cohort. These results align with previous studies suggesting that an incomplete CoW impairs collateral flow, increasing the risk of cerebral ischemia during carotid clamping. The significant differences in ICA stump pressure values between certain CoW groups (e.g., CoW 1 and 4, CoW 1 and 5) underscore the importance of preoperative CoW assessment in surgical planning and risk stratification. Despite comparable systemic hemodynamic status across CoW groups, the observed differences in ICA stump pressures highlight the intrinsic role of cerebral vascular anatomy in maintaining adequate perfusion. This finding is particularly relevant for surgical decision-making, as it underscores the potential necessity for shunting in patients with compromised CoW configurations. Our data support the practice of selective shunting based on preoperative CoW imaging, which can help mitigate perioperative neurological risks. Despite its utility, stump pressure measurement has some limitations. It offers a static snapshot of perfusion rather than a dynamic assessment, and its interpretation can vary based on patient-specific factors and the presence of contralateral carotid disease. Additionally, there is no universally accepted cutoff value for stump pressure that reliably predicts the need for shunting. Therefore, while stump pressure measurement is a cost-effective and widely used method, it is best utilized in conjunction with indices to ensure comprehensive intraoperative assessment and optimal patient outcomes.

The analysis of pulse pressure waveform areas further corroborates the significance of CoW integrity. Lower systolic, diastolic, and total areas under the pressure curve in patients with hypoplastic or isolated CoW variants suggest reduced interhemispheric blood flow compensation capacity.

The subjective evaluation of ICA stump backflow, classified into three patterns (strong pulsatile, continuous but not pulsatile, and weak discontinuous), showed a significant correlation with preoperative CoW imaging. Consistent with the blood pressure data, surgeons noted weaker ICA stump backflow in instances where the posterior CoW was incomplete and/or the MCA was isolated. Accordingly, moderate to weak ICA backflows were observed in cases where the anterior and/or posterior CoW semicircles were compromised. This relationship indicates that real-time intraoperative assessments can provide valuable supplementary information to preoperative imaging, aiding in the identification of patients at higher risk of compromised cerebral perfusion. We found that ICA pressure measurements showed low, non-zero pressures in the ICA stumps of the totally isolated MCA group, with consistently weaker backflow. The presence of stump pressure variation and similar diastolic pressure decays in different anatomical CoW variants, along with observed backflow in the clamped ICA stumps of the isolated MCA group, may act as vital intraoperative indicators. These factors might collectively signify the presence of a collateral system that supplements the compromised CoW circulation.

Our study also examined the relationship between contralateral carotid stenosis and pulse waveform variables. The weak correlations observed suggest that contralateral carotid pathology has a limited impact on intraoperative hemodynamics compared to CoW anatomy. This finding emphasizes the predominance of intrinsic cerebral vascular architecture over extracranial factors in influencing intraoperative cerebral perfusion during CEA.

Decreasing stump pressures and backflows from groups CoW 1 to CoW 5 suggest a diminished or absent pressure-equalizing function of these CoW configurations. Our hypothesis posits that the CoW’s blood storage volume is largest in a complete CoW and smallest in cases of isolated MCA. Consequently, a unit pressure change (Δ*P*) would introduce a smaller blood volume (Δ*V*) into a compromised CoW compared to a complete CoW, leading to a reduced storage capacity (compliance ~ C) of the cerebrovascular bed distal to the ICA, given that compliance (C) is defined as Δ*V*/Δ*P*. Using a two-element Windkessel model (which comprises cerebrovascular compliance and resistance elements) applied to the diastolic segment of a pressure waveform, the pressure decay time constant (τ) can be calculated. The finding that the diastolic pressure decay constant (τ) did not differ significantly among CoW groups suggests that the intrinsic autoregulatory mechanisms of the cerebral vasculature were similarly affected by systemic atherosclerotic disease in CAS patients, regardless of the anatomical configurations of the CoW.

In conclusion, our study highlights the robustness and reproducibility of our CTA protocol in evaluating the CoW morphology and guiding surgical decisions during CEA. The alignment of CTA-assessed CoW anatomy with intraoperative backflow measurements underscores the congruence between preoperative imaging and intraoperative observations, a finding further substantiated by the absence of neurological events in our cases. Implementation of a comprehensive preoperative assessment using CTA to evaluate the CoW is crucial for informed surgical decision-making, especially in determining the need for selective shunting and stent placement. Our approach, leveraging high-quality CTA imaging that accurately delineates the CoW, is validated by the semi-quantitative method and pulse wave measurement analysis. However, it is important to acknowledge that we could not establish a control group for ethical reasons. Despite this limitation, the confluence of our CTA findings with the intraoperative data offers a more confident framework for decision-making in CEA procedures.

Our study emphasizes the significant role of CoW in influencing ICA stump pressure waveform metrics during CEA. However, some limitations should be noted. The current study focuses primarily on perioperative outcomes and does not provide long-term follow-up data. We recognize that a compromised circle of Willis (CoW) may have lasting impacts on ischemic complications. In support of this, the work by Van Seeters et al.[74] has shown that patients with incomplete anterior CoW configurations are at significantly higher risk for anterior circulation strokes. Additionally, those with both anterior and posterior incompleteness face the highest risk of future ischemic events. These findings highlight the importance of long-term monitoring and management in patients with CoW anomalies, which aligns with our ongoing efforts to assess both immediate and long-term cerebrovascular outcomes in this cohort. To address this, our research group has been collecting longitudinal cognitive data using the Mini-Mental State Examination (MMSE) and Montreal Cognitive Assessment (MoCA) tests preoperatively, postoperatively, and during follow-up at 3 months and 1 year. Additionally, 2-year follow-up diffusion tensor imaging (DTI) MRIs were conducted. These data are currently being analyzed, and we plan to publish the findings in future manuscripts.

Furthermore, the classification of ICA stump backflow by surgeons may introduce some subjectivity, potentially affecting the consistency of key measurement. To minimize bias, all surgeons involved in the study underwent standardized training using predefined criteria for categorization (weak/discontinuous, continuous/non-pulsatile, and strong/pulsatile). This training was validated through pre-study pilot assessments and compared with objective measures, such as pulse pressure values, to ensure consistency in the evaluations. As reported in our results, the classifications of backflow were correlated with preoperative imaging findings of CoW morphology, yielding a Spearman correlation coefficient of 0.37. This moderate correlation demonstrates alignment between the subjective assessments and objective imaging data, supporting the validity of the classifications made during surgery. Additionally, intraoperative hemodynamic measurements, including ICA stump pulse pressure, were recorded to provide supplementary validation. The alignment between real-time assessments and objective data further emphasizes the utility of these classifications in guiding clinical decisions. While some subjectivity remains, the combination of subjective and objective data mitigates potential bias and allows for more informed intraoperative decision-making.

Lastly, this study is limited to asymptomatic patients undergoing carotid endarterectomy (CEA) for carotid artery stenosis (CAS), which may restrict the broader applicability of the findings to other cerebrovascular conditions or procedures. However, the hemodynamic principles observed—such as the relationship between ICA stump pressure, backflow, and CoW morphology—are not unique to CEA. The CoW plays a crucial role as a collateral pathway in maintaining cerebral perfusion across various cerebrovascular conditions, including stroke and intracranial atherosclerosis. The established link between CoW completeness and collateral circulation in conditions like ischemic stroke underscores the broader relevance of our findings. Although the study focuses on CEA patients, the observation that incomplete CoW anatomy is associated with impaired hemodynamics could also apply to other vascular occlusions and interventions aimed at restoring cerebral perfusion. Furthermore, intraoperative measurements such as stump pressure and backflow reflect fundamental cerebrovascular dynamics that are likely transferable to interventions such as intracranial stenting or management of large vessel occlusions.

Our findings suggest that comparable diastolic time constants in different CoW anatomies could indicate a protective capillary collateral system in impaired CoW scenarios, potentially safeguarding against perioperative neurovascular incidents. This underscores the need for further research to quantify this protective effect. Furthermore, despite the subjective nature of assessing ICA backflow by the surgeon, it provides valuable insights, particularly in understanding the operator’s role in stent application. By integrating thorough preoperative CoW assessments with intraoperative data, we were able to refine surgical approaches, thereby improving patient outcomes and safety in CEA procedures. These results not only enhance our understanding of key factors influencing the success and potential complications of CEA but also highlight the critical role of advanced imaging techniques in improving surgical outcomes. The integration of detailed preoperative CoW imaging with intraoperative monitoring can enhance the identification of patients at risk for perioperative neurological events, guiding the use of selective shunting and improving surgical outcomes. Future studies should aim to refine these techniques and explore additional factors influencing cerebral perfusion in patients with CAS, particularly the impact of systemic atherosclerotic vascular disease on cerebral small vessel disease (CSVD) [75, 76], to further optimize patient care in carotid artery surgery. Systemic atherosclerosis can lead to cerebromicrovascular endothelial dysfunction, characterized by a reduced ability of blood vessels to dilate. This endothelial dysfunction, coupled with microvascular rarefaction and pathological remodeling of resistance, can further compromise interhemispheric blood flow compensation. Understanding these additional factors is crucial for developing comprehensive treatment strategies to optimize cerebral perfusion and minimize the risk of perioperative neurological complications in CAS patients.

## Data Availability

All data generated or analyzed during this study are included in this published article (and its supplementary information files). Due to the sensitive nature of the individual patient data, full data cannot be made openly available. De-identified data that underpin the findings are, however, available on reasonable request to the corresponding author. Any additional data related to this study is available from the corresponding author on reasonable request. This is to comply with data privacy regulations while ensuring that the data necessary for replicating this study’s findings are readily available to those who require it. Please note that any request will be subject to a data use agreement to ensure the confidentiality and privacy of the data used is maintained.

## Abbreviations

CoW: Circle of Willis
CEA: Carotid endarterectomy
CTA: Computed tomography angiography
ICA: Internal carotid artery
MRI: Magnetic resonance imaging
ANOVA: Analysis of variance
DSA: Digital subtraction angiography
TCD: Transcranial Doppler
CT: Computed tomography
MR: Magnetic resonance
NIRS: Near-infrared spectroscopy
EEG: Electroencephalography
SSEP: Somatosensory evoked potentials
TIA: Transient ischemic attack
ICD: Implantable cardioverter defibrillator
AHA: American Heart Association
ESC: European Society of Cardiology
ESVS: European Society for Vascular Surgery
BMT: Best medical therapy
NASCET: North American Symptomatic Carotid Endarterectomy Trial
IMR: Iterative model reconstruction
A-com: Anterior communicating artery
A1: First segment of the anterior cerebral artery
P-com: Posterior communicating artery
P1: First segment of the posterior cerebral artery
MCA: Middle cerebral artery
DTI: Diffusion tensor imaging
FLAIR: Fluid-attenuated inversion recovery
SWI: Susceptibility-weighted imaging
DWI: Diffusion-weighted imaging
CCA: Common carotid artery
ECA: External carotid artery
ECG: Electrocardiogram
